# Feasibility study on a digital version of Good Psychiatric Management delivered in routine psychiatric care

**DOI:** 10.1016/j.invent.2026.100937

**Published:** 2026-03-24

**Authors:** Dan Bengtsson, Hanna Sahlin, Viktor Kaldo, Fredrik Falkenström

**Affiliations:** a Department of Psychology, Faculty of Health and Life Sciences, Linnaeus University, Växjö, Sweden; b Centre for Psychiatry Research, Department of Clinical Neuroscience, Karolinska Institutet, Region Stockholm, Sweden; c Stockholm Health Care Services, Region Stockholm, Sweden

**Keywords:** Good Psychiatric Management, Personality disorders, Personality difficulties, ICD-11, digital intervention, Guided self-help, Feasibility, Acceptability, Psychiatric outpatient care, Negative effects

## Abstract

**Background:**

Scalable, low-threshold interventions for personality disorders and personality difficulties (ICD-11) remain limited. We developed GPM-I, a guided digital self-help adaptation of Good Psychiatric Management, to deliver structured psychoeducation and support early change efforts.

**Methods:**

We conducted a naturalistic feasibility study in routine psychiatric care in Stockholm, Sweden. Feasibility outcomes included engagement and standard program completion (Modules 1–2) and optional advanced module uptake (Modules 3–6). Acceptability was assessed with the Client Satisfaction Questionnaire (CSQ-8) and negative effects with the Negative Effects Questionnaire (NEQ). Weekly distress (CORE-10) was analyzed using linear mixed-effects models.

**Results:**

Of 131 patients offered access, 78 (59.5%) initiated the program by completing Module 1 (engagement threshold). Participants were 71.8% women; mean age was 35.2 years. Participants were categorized as having personality disorder (*n* = 18), personality difficulties (*n* = 22), or other diagnoses (*n* = 38). Among initiators, standard program completion (Modules 1–2) was observed in 40/78 participants (51.3%), and 13/78 (16.7%) completed all available modules (Modules 1–6). CSQ-8 respondents (28/78, 35.9%) reported moderate satisfaction, with 85.7% rating overall satisfaction in the two highest categories, but completion of CSQ-8 and NEQ was low. Negative effects were reported by 25/27 NEQ respondents (92.6%), most often increased stress/pressure or emotional strain. CORE-10 decreased significantly over time with a small effect size.

**Conclusion:**

GPM-I showed moderate engagement and moderate acceptability among respondents, alongside generally mild-to-moderate negative effects. Findings support continued refinement of the procedures and can inform the design of a controlled trial.

## Highlights

• A guided digital version of Good Psychiatric Management was evaluated.
• Included patients with ICD-11 personality disorder and personality difficulty
• Over half of invited patients initiated the program.
• Acceptability was high and negative effects were uncommon.
• Symptom change was modest and exploratory.

## 1 Introduction

Digital delivery is increasingly central in psychiatric care and can, when delivered as guided digital self-help with structured material that patients work through independently, supported by brief clinician contact, yield effects comparable to face-to-face treatment while extending access to populations who might not otherwise access care (Andersson et al., 2014; Carlbring et al., 2018; Păsărelu et al., 2017). In routine outpatient psychiatry, many patients present with clinically significant personality-related difficulties that contribute to functional impairment and recurrent service use, yet they do not qualify for specialized personality disorder (PD) programs. At the same time, their needs often exceed what low-intensity or symptom-specific interventions can reasonably address, creating a common service gap in structured care. Robust evidence exists for internet-delivered interventions for common mental health problems, and digital programs can also reach individuals who may not engage in conventional services (Andersson et al., 2019a; Mamukashvili-Delau et al., 2022).

However, questions remain about implementation in routine services and in diagnostically complex groups (Andersson et al., 2019b; Xie et al., 2022). For personality pathology specifically, early work indicates promise for structured digital formats, including moderate effects in programs targeting borderline personality disorder and benefits from components such as self-monitoring and behavioral rehearsal, although evidence for their effectiveness in routine psychiatric care is still emerging (Drews-Windeck et al., 2023; Hudon et al., 2022; Lindsay et al., 2024).

Personality disorders (PD) are common and disabling conditions characterized by impairments in self- and interpersonal functioning (World Health Organization, 2019). They are associated with substantial individual suffering and societal burden, including impaired social and vocational functioning (Leichsenring et al., 2011; Zanarini et al., 2005). Within psychiatric services, their centrality is reflected in prevalence estimates approaching half of outpatient samples (Beckwith et al., 2014). Contemporary diagnostic nosology in major health care systems emphasizes a dimensional approach to personality pathology, prioritizing level of personality functioning and severity rather than categorical subtypes (Hopwood et al., 2013; Tyrer et al., 2011). The International Classification of Diseases, 11th Revision (ICD-11), formalizes a severity continuum from personality difficulties to mild, moderate, and severe PD, anchored in impairments in self- and interpersonal functioning, with the threshold for a PD diagnosis set at the level of mild severity (WHO, 2019). Even subthreshold impairments, termed personality difficulties, can meaningfully disrupt everyday functioning and social participation, broadening the range of clinically relevant presentations and shaping treatment planning (Hopwood et al., 2013; Tyrer et al., 2019). For example, a patient may not meet criteria for a PD diagnosis yet show persistent impairments in self- and interpersonal functioning that contribute to recurrent relational breakdowns, occupational instability, and elevated service use.

Despite this reframing, evaluated interventions still concentrate on borderline personality disorder, and evidence for other PDs and for subthreshold presentations remains scarce, contributing to a treatment gap in routine services (Katakis et al., 2023). Evidence-based treatments for PDs, including approaches such as dialectical behavior therapy (DBT) and mentalization-based therapy (MBT), are effective but lengthy, complex, and difficult to scale to routine clinical services (McMain et al., 2018). Many patients with personality difficulties or milder personality pathology do not qualify for specialist programs, yet present with emotion dysregulation, interpersonal problems, and functional impairment that warrant structured help, underscoring the need for scalable, lower-intensity options aligned with a dimensional and transdiagnostic view (Bach et al., 2022; Hutsebaut et al., 2020; Natoli et al., 2024). At the same time, digital modalities can reduce barriers to timely support, but their implementation for personality-related problems in routine care has received limited empirical attention (Xie et al., 2022). Taken together, ICD-11's broader clinical target group and routine-care constraints highlight the need for pragmatic, scalable interventions that can provide structured orientation, psychoeducation, and support for attainable functional change goals without requiring lengthy specialist treatments. Generalist models designed for routine care may be particularly well suited to this role.

### 1.1 Good Psychiatric Management

Generalist, pragmatic models may help close this gap. Good Psychiatric Management (GPM) (Choi-Kain et al., 2016; Gunderson and Links, 2014) provides a structured, clinician-friendly approach developed for borderline PD that integrates psychoeducation, interpersonal focus, active case management, and the pursuit of attainable functional goals within a biopsychosocial frame. In a randomized controlled trial, GPM showed outcomes comparable to DBT (McMain et al., 2009), and later reviews suggest that GPM can function as a scalable option in routine services (Choi-Kain et al., 2016; Gunderson et al., 2018). Accordingly, GPM was developed to support wider dissemination of structured, evidence-informed within routine care and to facilitate delivery by generalist clinicians (Choi-Kain et al., 2016; Gunderson et al., 2018; Javaid et al., 2025). The model's structure and focus on everyday functioning make it amenable to digital adaptation and potentially suitable for patients whose difficulties are clinically significant yet fall below thresholds for specialized treatments (Choi-Kain et al., 2016).

Although GPM was developed for BPD, it is framed as a generalist, principle-based model that structures routine care around psychoeducation, an interpersonal focus, pragmatic case management, and attainable functional change goals. These targets are not BPD-specific and map closely onto ICD-11's definition of personality pathology, which centers on impairments in self- and interpersonal functioning across a severity continuum (WHO, 2019). On this basis, GPM provides a scalable framework that may be applicable across personality presentations when the shared clinical need is structured orientation and support for functional change. Consistent with this view, recent clinical work has described the use of GPM-informed conceptualizations in dimensional assessment and broader personality disorder presentations (Blay et al., 2023).

### 1.2 Digital adaptation: GPM-I

To combine the pragmatic strengths of generalist approaches with the opportunities offered by digital delivery, we created a digital version of GPM (GPM-I). GPM-I refers to a digital, Internet-delivered version of Good Psychiatric Management. The “I” denotes its Internet-based delivery format, although we use the broader term “digital” throughout the article to reflect contemporary terminology. Delivered as guided self-help organized into brief, structured modules that combine psychoeducational text, reflection exercises, and practical tools, the program offers material that patients work through independently with weekly clinician support. It serves to address this treatment gap and provide low-threshold interventions for individuals with milder personality pathology or personality difficulties.

It was designed as a low-threshold, guided self-help intervention in which clinicians support the patient's work with the digital self-help material, targeting individuals with personality difficulties or PD according to ICD-11. The program consists of digital self-help material that focuses on two core functions central to GPM: providing psychoeducation to help patients recognize personality-related mechanisms behind their presenting problems, and offering structured support to initiate concrete change efforts in everyday life domains such as relationships, work or study, health and daily routines (Choi-Kain et al., 2016; Gunderson and Links, 2014). In our service context, this format aims to shorten the distance between assessment and action by providing orientation and tools that patients can use immediately rather than remaining without structured support while awaiting further clarification, next-step planning, or additional interventions within routine psychiatric care.

### 1.3 Rationale

The rationale behind GPM-I is that by addressing personality-related problems, such as personality disorder, personality difficulties, and personality-related problems occurring alongside other primary psychiatric conditions, services may reach patients earlier and provide psychoeducation together with basic support for initiating change goals. Informed by the ICD-11 dimensional model and the generalist framework of GPM, the intervention was designed to combine orientation and education with practical steps toward change, adapted for delivery within Psykiatri Sydväst, Stockholm, Sweden, by the Personality Disorders Unit.

### 1.4 Aims

The present open feasibility study examined the implementation of GPM-I within regular psychiatric outpatient services. The primary aim was to evaluate feasibility and acceptability, operationalized as adherence, engagement, and completion, client satisfaction, negative effects, recruitment source, and adherence to the assessment procedures (Attkisson and Zwick, 1982; Rozental et al., 2016). Exploratory outcomes included pre-to-post symptom change on routinely used measures, treated as signal data to inform a subsequent randomized evaluation (Barkham et al., 2013; Bjureberg et al., 2016; Bohus et al., 2009; EuroQol Group, 1990; Kroenke et al., 2001; Spitzer et al., 2006; Ustün et al., 2010). A secondary exploratory aim was to examine whether feasibility indicators differed across clinical pathways and diagnostic groups and whether module completion differed between participants with and without a diagnosed personality disorder. By positioning feasibility and acceptability as the primary outcomes, with symptom change analyzed as signal data, the study aligns with the service need to determine whether such a format can be pragmatically delivered and received by patients whose personality-related difficulties often place them between conventional treatment pathways (Andersson et al., 2019a; Hedman-Lagerlöf et al., 2023; Katakis et al., 2023).

## 2 Method

This study employed a naturalistic, prospective open design with a primary focus on feasibility and acceptability. Given the early implementation stage and the primary aim of assessing feasibility and acceptability in routine care, we did not attempt randomization or a comparator condition; controlled evaluation is planned as a subsequent step. Symptom change was examined as a secondary outcome. GPM-I was evaluated in adults categorized into three diagnostic groups (Personality Disorder [PD], Personality Difficulties [PSP], and Mixed Diagnostic Group [MDG]; defined in the Participants section). Participants were recruited through three clinical pathways described in the Recruitment subsection, and these pathways were examined alongside diagnostic groups in exploratory analyses. We used a pre-post design with continuous weekly assessments to explore changes in psychological distress and other relevant outcome measures over the course of the intervention.

### 2.1 Ethical approval and consent

The study was conducted in accordance with ethical guidelines and approved by the Regional Ethical Review Board in Sweden (dnr 2022-06382-01). All participants provided written informed consent prior to inclusion.

### 2.2 Context, recruitment, and participants

Participants were recruited between August 2023 and December 2024 from outpatient psychiatric services at Psykiatri Sydväst, Huddinge, Stockholm. Potential participants were identified through three pathways, described in the next subsection. In all pathways, clinicians introduced GPM-I to patients for whom personality-related problems – ranging from suspected to established personality disorder – were judged clinically relevant. Patients received brief oral information together with a written information pamphlet and were invited to start the program if both patient and clinician agreed that the format was appropriate. The inclusion and exclusion criteria listed below guided, but did not solely determine, this joint decision. A brief overview of these recruitment pathways is provided in the next subsection, where each pathway is described in detail.

#### 2.2.1 Clinical pathways and recruitment flows

##### 2.2.1.1 Overview of clinical pathways

To clarify how patients entered GPM-I in routine psychiatric care within Psykiatri Sydväst, where patient flow follows the organizational structure of the local service, we classified recruitment into three main clinical pathways based on unit of origin. In brief, Pathway A comprised patients undergoing initial psychiatric assessment at the Assessment Unit (UE), Pathway B comprised patients already in care within neuropsychiatric services (ADHD/NP units), and Pathway C comprised patients assessed at the Personality Disorders Unit (PB) who were awaiting specialized treatment. This classification reflects how patients typically move through the service and captures the most common organizational routes by which individuals were offered the intervention. The pathways reflect organizational entry points, whereas diagnostic groups (Personality Disorder [PD], Personality Difficulties [PSP], and Mixed Diagnostic Group [MDG]) were defined separately and used exclusively for analytic purposes (with detailed descriptions provided in the Diagnostic Groups section). Across pathways, GPM-I was delivered in different care contexts, either as a stand-alone, low-threshold intervention during assessment or while awaiting further services, or as an adjunct to ongoing psychiatric care; concurrent care was not standardized and reflected routine service delivery.

##### 2.2.1.2 Pathway A: Assessment Unit (UE) – initial psychiatric assessment

This pathway represents patients who were newly referred to psychiatry and underwent a comprehensive evaluation at the Assessment Unit. Individuals in this pathway were assessed by their responsible clinician, who identified problems consistent with the personality domain and they were therefore offered GPM-I as an early, low-threshold intervention while awaiting further clarification or referral. This pathway reflects the use of GPM-I as an initial orientation to psychiatric care.

##### 2.2.1.3 Pathway B: neuropsychiatric services (ADHD/NP units)

Patients in this pathway were already receiving psychiatric care, usually within ADHD or neurodevelopmental disorder units (e.g. the ADHD-program). Although their primary diagnostic focus was ADHD or ASD, many presented with emotion regulation problems, interpersonal difficulties, or functional impairments consistent with clinically relevant personality difficulties. GPM-I was offered as an adjunctive intervention to support these difficulties in parallel with ongoing treatment for the primary condition. This pathway represents a more stable group whose main contact remained within their regular unit.

##### 2.2.1.4 Pathway C: Personality Disorders Unit (PB) – pre-treatment/waitlist group

This pathway included patients who had already undergone extended personality assessment and were awaiting specialized treatment for personality disorder, such as MBT or GPM-based group treatment. For these individuals, GPM-I functioned as a bridging intervention during the waiting period. This pathway typically reflects patients with more severe or established personality pathology who were already integrated into specialist services.

Additional, less frequent pathways existed (e.g. emergency services, Affective and Anxiety Disorders Program, Internet Psychiatry Unit), but these did not represent stable organizational routes and were therefore not defined as separate pathways.

#### 2.2.2 Participant assessment and diagnostic classification

All participants underwent comprehensive psychiatric assessment conducted by regular clinical staff, establishing their primary diagnosis. Primary diagnoses were established by psychiatrists at the Assessment Unit (UE). For suspected PD, additional evaluation was conducted at the specialized Personality Disorders Unit by clinicians with training in PD diagnostics, using standardized assessment interviews (e.g. SCID-II, STiP-5). All participants in the PD group underwent such extended assessments, whereas those in the PSP and MDG groups did not. Classification of personality difficulties below the diagnostic threshold followed the ICD-11 dimensional framework (WHO, 2019), focusing on impairments in self-functioning and interpersonal functioning. To support consistency in applying ICD-11 personality difficulty criteria in routine care, clinicians relied on a shared framework anchored in ICD-11 self- and interpersonal functioning. Classifications were discussed as needed in team supervision/case discussions.

#### 2.2.3 Diagnostic groups

Participants were categorized into three diagnostic groups: (1) Personality Disorders (PD): participants meeting full diagnostic criteria for one or more PDs; (2) Personality Difficulties (PSP): participants with clinically significant personality difficulties consistent with ICD-11 personality disorder, but not meeting the full diagnostic criteria for PD; and (3) Mixed Diagnostic Group (MDG): participants with a primary diagnosis other than PD (primarily neurodevelopmental, anxiety or affective disorders) who were judged by their treating clinician, and confirmed by the patient, to have clinically significant personality difficulties. This classification reflects routine psychiatric care, where personality-related difficulties commonly co-occur with other primary diagnoses and may not be captured as the primary diagnostic label. Accordingly, feasibility specific to patients primarily presenting with personality pathology cannot be established from the MDG subgroup alone and is interpreted separately from the PD/PSP subgroups.

#### 2.2.4 Inclusion and exclusion criteria

The inclusion criteria required participants to be adults (≥18 years) with sufficient Swedish reading and writing ability to engage with the digital materials. All potential participants underwent an information session by telephone, to ensure they had a realistic appreciation of GPM-I, including appropriate expectations about the scope and goals of the intervention. Eligibility was assessed against the predefined criteria by a clinician at the Personality Disorders Unit; the clinician made the final inclusion decision and documented eligibility and inclusion in the medical record.

Predefined exclusion criteria focused on acute psychiatric states that would preclude meaningful engagement or require more intensive intervention (e.g., untreated mania, active psychosis, or severe psychiatric impairment requiring immediate clinical attention). We also defined concurrent participation in other specialized psychological treatments for personality disorders as exclusionary. However, during the study period no participants met these criteria, meaning that all individuals who provided informed consent and fulfilled the inclusion criteria were offered participation.

### 2.3 Intervention: background, adaptation, and content

GPM-I is a digital adaptation of Good Psychiatric Management (GPM) developed at the Personality Disorder Unit at Huddinge University Hospital in Sweden. The intervention was based on the principles of GPM (Gunderson et al., 2018) and adapted to the clinical setting using an unpublished manual (Bengtsson, 2021). GPM-I aimed to increase accessibility while retaining the core therapeutic elements of GPM.

GPM-I was delivered as a structured, guided self-help via Sweden's national digital platform, Stöd och Behandling (SoB). Participants accessed the material on a computer, tablet or smartphone using secure electronic identification (BankID). The program consisted primarily of self-help content that patients worked through independently. Each module contained psychoeducational text, structured reflection exercises, and practical tools designed to be applied in daily life. A substantial proportion of the work involved engaging with the self-help material on the platform, with the exercises designed to be applied in daily life. Participants were encouraged to try out ideas and reflect between logins, but the core of the intervention remained the guided digital self-help format. Therapist support was provided through brief, weekly written feedback focused on engagement with the self-help material, clarification of module content, and supporting the application of exercises in everyday situations. Participants also initiated contact with their therapists through the platform when needed.

An overview of the modules is provided in Table 1. Module 1 was more extensive than the subsequent modules and provided a condensed version of the key elements of the program. The standard program comprised Modules 1 and 2, which contained the essential intervention content, presented in shorter and more accessible form. Module 1 was the most content-rich, offering a concentrated version of GPM's fundamental principles, while Modules 3–6 were optional advanced modules that provided extended material for participants wishing to deepen their work beyond the standard program. Completion of the standard program was defined as completing at least two modules (Modules 1–2).

**Table 1:** Overview of GPM-I modules and content.

Module	Title	Brief description
1	Introduction and Core Concepts (Standard program)	Psychoeducation on personality-related problems, conflicts between emotions and goals, and balance.
2	Change and Motivation (Standard program)	Overcoming avoidance and building toward meaningful change.
3	Exploring Emotions (Optional advanced)	Identifying, understanding, and expressing emotions.
4	Emotion Regulation (Optional advanced)	Strategies for tolerating and managing difficult emotions.
5	Self-Image and Identity (Optional advanced)	Reflection on self-concept, values, and interpersonal roles.
6	Tools and Framework (Optional advanced)	Summary of key strategies, relapse prevention, and planning for continued use.

The program was designed to be flexible and accessible, with each module comprising 7–10 chapters of approximately 200–400 words each, combining psychoeducational text, reflective exercises, and practical tools. Participants were encouraged to complete one module per week, typically in two to three study periods of 15 to 20 min. They were also encouraged to apply the material to their daily lives and align it with personal goals. Therapist feedback supported engagement and individualized adaptation throughout the program.

Feedback was typically provided once per week by psychologists at the Personality Disorders Unit, all with clinical experience of personality disorders and training in GPM. The time spent on each participant varied between approximately 10 and 40 min per week, depending on how much the participant engaged with the material. All feedback was delivered in writing, and the frequency could increase when participants initiated additional contact.

The core content was concentrated in Module 1 and Module 2, which covered personality-related problems, emotional processes, balance across life domains, and therapeutic goal setting.

### 2.4 Measures

Data were collected at baseline (pre), weekly throughout the intervention, and at posttreatment. Baseline and posttreatment assessments captured feasibility, acceptability, and exploratory symptom outcomes, while weekly assessments indexed changes in psychological distress over time.

Prior to starting GPM-I, participants completed the program's baseline self-report assessment (pre), consisting of standardized instruments to evaluate psychological functioning. Throughout the intervention, participants completed weekly assessments to monitor psychological distress. At posttreatment, participants completed the same measures as at baseline plus additional measures described below. CSQ-8 and NEQ were intended for all participants at posttreatment; however, because these questionnaires were not embedded as required steps in the platform workflow, they could be skipped, contributing to lower response rates. These outcomes are therefore reported descriptively for responders.

Non-inclusion in the feasibility analyses resulted either from being non-starters (i.e., participants who did not complete the PRE-assessment and therefore never engaged with the program) or from being dropouts (i.e., participants who completed the PRE-assessment and initiated activity but did not complete Module 1). Participants who completed Module 1 were classified as initiators, with standard program completion defined as completion of Modules 1–2.

#### 2.4.1 Outcome measures

The assessment battery was aligned with existing clinical procedures at the Personality Disorders Unit, reflecting routine practice and the established routine for administering these measures. These standardized and validated instruments facilitated clinical benchmarking and supported ecological validity and interpretability, with feasibility endpoints designated as the primary outcomes. Measures were selected to minimize participant burden, leverage staff familiarity, and enable comparability with routinely collected service data. The battery covered key domains relevant to personality-related impairment and common comorbidity (distress, emotion regulation, depression/anxiety, functioning, and quality of life), and scores were interpreted as indices of symptom severity and functioning rather than as stand-alone diagnostic indicators.

##### 2.4.1.1 Primary outcomes (feasibility and acceptability)

Module adherence and completion. Adherence was operationalized as the number of completed modules (0–6). Completion of the standard program was defined as completing at least two modules (Modules 1–2). Completion of Module 1 was defined as the minimum threshold for initiation and was used to categorize participants into non-starters, dropouts, and initiators.

##### 2.4.1.2 Patient satisfaction

Satisfaction with the intervention was measured using the Client Satisfaction Questionnaire–8 (CSQ–8; Attkisson and Zwick, 1982). The scale yields a total score ranging from 8 to 32, with higher values indicating greater satisfaction. It was administered at posttreatment.

##### 2.4.1.3 Negative effects

Adverse or unwanted experiences were assessed with the Negative Effects Questionnaire (NEQ; Rozental et al., 2016), which provides an index of negative events attributed to psychological interventions. The total/impact score was used, and the measure was administered at posttreatment.

##### 2.4.1.4 Recruitment source and assessment adherence

Recruitment source was defined as the clinical unit from which each participant originated. Assessment adherence captured completion of posttreatment feasibility measures (CSQ–8 and NEQ) and weekly assessment participation throughout the intervention.

##### 2.4.1.5 Secondary outcomes

The Clinical Outcomes in Routine Evaluation-10 (CORE-10; Barkham et al., 2013) is a 10-item self-report measure of global psychological distress, including symptoms of depression, anxiety, and impaired functioning. It has demonstrated good internal consistency (α > 0.80) and sensitivity to change in clinical populations (Barkham et al., 2013). Scores were reported as item means on the 0–4 scale, consistent with the scoring used in the present service evaluation.

The Borderline Symptom List-23 (BSL-23; Bohus et al., 2009) assessed borderline-related symptoms. Emotion regulation difficulties were measured with the Difficulties in Emotion Regulation Scale–16 (DERS-16; Bjureberg et al., 2016). Emotion regulation strategies were additionally assessed with the Emotion Regulation Questionnaire (ERQ; Gross and John, 2003) on a pre–post basis.

To provide a broader picture of participants' mental health and functioning, we included the following measures. The Patient Health Questionnaire-9 (PHQ-9; Kroenke et al., 2001) assessed depressive symptoms and has shown high reliability and criterion validity. The Generalized Anxiety Disorder-7 (GAD-7; Spitzer et al., 2006) measured symptoms of generalized anxiety. Functional impairment was assessed with the World Health Organization Disability Assessment Schedule 2.0 (WHODAS 2.0; Ustün et al., 2010). The EuroQol–5 Dimensions (EQ-5D; EuroQol Group, 1990) provided a standardized index of health-related quality of life.

### 2.5 Statistical analysis

Given the feasibility design, all inferential analyses were exploratory and intended to describe patterns rather than provide confirmatory tests of effectiveness. All analyses were conducted in R (Version 4.3.1). Primary analyses summarized feasibility and acceptability using descriptive statistics (frequencies, percentages, M, SD, ranges) for module completion, CSQ–8, NEQ, recruitment source, and assessment adherence. Engagement was defined as completion of Module 1. Intervention adherence was summarized using the number of completed modules (0–6) and standard program completion, defined as completing at least two modules (Modules 1–2). Exploratory comparisons of standard program completion between participants with and without a PD diagnosis were conducted using two–tailed Mann–Whitney *U* tests.

Secondary, exploratory pre-post outcomes were summarized descriptively per instrument (means, SDs, mean change, Cohen's d with 95% CIs). Analyses were conducted pairwise, so that only participants with both pre- and post-treatment data on a given instrument were included in that instrument's analysis. All inferential analyses were two-tailed with *α* = 0.05.

Weekly CORE–10 scores were analyzed using a linear mixed-effects model (random intercepts and slopes for participants; log-transformed time as a fixed effect), estimated via maximum likelihood. Model comparison was based on AIC. Mixed-effects models were fitted with the *lme4* package.

## 3 Results

Results are presented to distinguish feasibility outcomes from exploratory clinical outcomes. We first report participant flow, engagement, and module completion patterns, followed by acceptability and negative effects. We then present exploratory symptom outcomes and the longitudinal CORE-10 analysis to describe change patterns over time, without interpreting these findings as confirmatory effectiveness results.

### 3.1 Participant characteristics and missing data

**Fig. 1:**
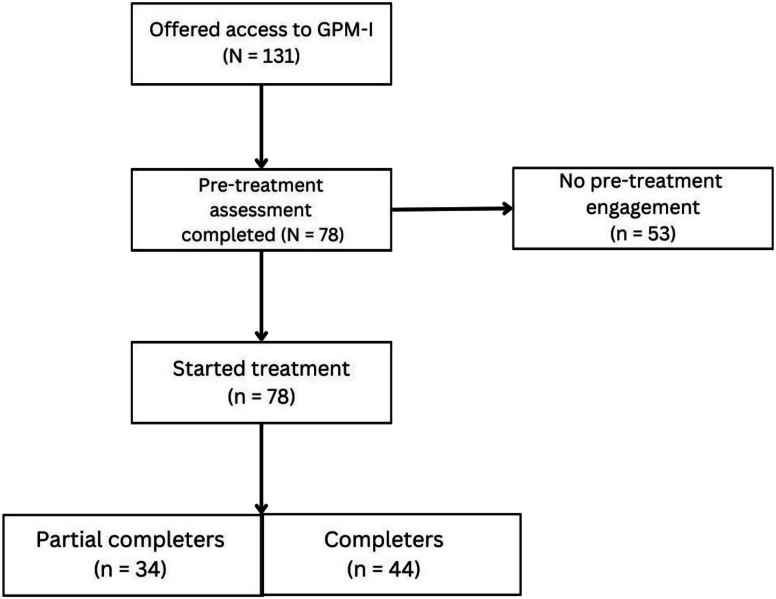
Participant flow diagram. *Note*. Of the 131 individuals offered access to GPM-I, 78 completed the pre-treatment assessment and subsequently initiated the program by completing Module 1. “No pre-treatment engagement” refers to participants who did not complete any pre-treatment measures or initiate the program (i.e., did not complete Module 1). Standard program completion was defined as completion of Modules 1–2.

Of the 131 participants recruited, 78 engaged with the program by completing Module 1 (the engagement threshold). Assessment provision for these participants is summarized in Fig. 1. There was substantial variation in posttreatment assessment completion, with higher missingness at post compared to baseline. Details are presented in the flow diagram (Fig. 1) and in the respective outcome tables. No participants were excluded based on the predefined criteria during the study period. A small number of participants were recruited from other units (emergency services, the Affective and Anxiety Disorders Program, Internet Psychiatry; 2–4 per unit). Because these subgroups were too small to form meaningful categories, they were included only in the aggregated analyses.

Participant characteristics are summarized in Table 2. Participants were categorized into three diagnostic groups: PD (*n* = 18), PSP (*n* = 22), and MDG (*n* = 38). The sample included 56 females (71.8%) and 22 males (28.2%), with a mean age of 35.2 years (*SD* = 12.0; range = 20–78 years).

**Table 2:** Baseline characteristics of the sample (*N* = 78).

Characteristic	n (%) or M (SD)
Age (years)	35.2 (12.0), range 20–78
Gender	
Female	56 (71.8)
Male	22 (28.2)
Diagnostic group	
Personality Disorder (PD)	18 (23.1)
Personality Difficulties (PSP)	22 (28.2)
Mixed Diagnostic Group (MDG)	38 (48.7)
Program completion	
Completed standard program (Modules 1–2)	40 (51.3)
Initiators without standard completion	38 (48.7)

*Note*. Completion categories refer to initiators (*N* = 78). In the MDG, 25 participants had a neurodevelopmental disorder (20 ADHD, 5 AST), 7 had anxiety disorder, and 6 had an affective disorder.

### 3.2 Assessment completion and missing data

Among the 78 participants who engaged with the intervention, 59 (75.6%) provided at least one posttreatment measure, while 19 (24.4%) provided no posttreatment assessments. A flow diagram illustrating participant inclusion and module completion is presented in Fig. 1.

### 3.3 Feasibility and acceptability outcomes

#### 3.3.1 Module completion (standard program completion and optimal advanced module uptake)

Standard program completion (Modules 1–2) was observed in 40/78 participants (51.3%). On average, participants completed 2.54 modules (*SD* = 1.91; range = 1–6). Although 78 participants met the engagement threshold by completing Module 1, 38/78 (48.7%) did not complete Module 2, indicating a substantial drop-off after Module 1. Completion of all available modules (Modules 1–6) was observed in 13/78 participants (16.7%), indicating that uptake of the optional advanced modules (Modules 3–6) was lower overall (Table 3).

**Table 3:** Feasibility outcomes: adherence, acceptability, negative effects, and recruitment source (*N* = 78).

Outcome	n (%)/M (SD)
Adherence and completion	
Completed standard program (Modules 1–2)	40 (51.3)
Completed all available modules (Modules 1–6)	13 (16.7)
Mean number of modules completed	2.54 (1.91)

Acceptability (CSQ–8)	
Respondents	28 (35.9)
Total score (8–32), M (SD)	20.9 (1.47)
Mean item score (1–4), M (SD)	2.61 (0.18)
Overall satisfaction (upper two categories)	85.7%
Willingness to return (upper two categories)	78.6%
Helped deal more effectively (upper two categories)	67.9%

Negative Effects (NEQ)	
Respondents	27 (34.6)
Mean total severity (1–5), M (SD)	2.61 (0.68)
≥1 negative effect endorsed	25 (32.1)

Recruitment source	
Assessment Unit	38 (48.7)
ADHD Unit	21 (26.9)
Personality Disorders Unit	9 (11.5)
Emergency Outpatient Unit	4 (5.1)
Internet Psychiatry Unit	4 (5.1)
Anxiety & Affective Disorders Unit	2 (2.6)

*Note*. CSQ–8 = Client Satisfaction Questionnaire–8. NEQ = Negative Effects Questionnaire. Percentages are calculated based on the total sample (*N* = 78) unless otherwise indicated. Ranges for CSQ–8 (observed 18–24) and NEQ (1.00–3.85) reflect observed values in the sample. The standard program comprised Modules 1–2; Modules 3–6 were optional advanced modules, and completion of the standard program was defined as completing at least two modules (including participants who continued into optional modules).

There was no significant difference in standard program completion between participants with and without a PD diagnosis (*U* = 589.0, *p* = .32).

#### 3.3.2 Patient satisfaction

Posttreatment, 28 of 78 participants completed the CSQ-8 (35.9%). The mean total score was *M* = 20.9 (*SD* = 1.47; observed range = 18–24; possible range 8–32), corresponding to a mean item score of 2.61. On the item level, 85.7% rated overall satisfaction in the two highest categories, 78.6% indicated willingness to return for similar help, and 67.9% reported that the program helped them deal more effectively with their problems (see Table 3).

#### 3.3.3 Negative effects (open-ended responses)

The NEQ was completed by 27 participants (34.6%). Among respondents, 25/27 (92.6%) endorsed at least one negative effect. When expressed relative to all participants who initiated the program (*n* = 78), this corresponds to 25/78 (32.1%) and should be interpreted as a lower-bound estimate given the low response rate. Mean severity among respondents was *M* = 2.61 (*SD* = 0.68, range = 1.00–3.85). The proportion of respondents reporting at least one negative effect is presented in Table 3.

##### 3.3.3.1 Adverse events

Open-ended responses to the NEQ were provided by 16 participants. The most frequently reported adverse events were increased stress or pressure (*n* = 6) and emotional strain (*n* = 4). Less common categories included negative self-view or alliance issues (*n* = 1), dependence or difficulty after program end (*n* = 1), worry or sleep disturbance (*n* = 1), dropout due to negative experiences (*n* = 1), and technical or form issues (*n* = 1). Most participants who reported an adverse event explicitly indicated in their responses that it was caused, at least in part, by the intervention (see Table 3).

#### 3.3.4 Recruitment source

Participants originated from multiple units within the main clinic: The Assessment Unit (*n* = 38), the ADHD Unit (*n* = 21), the Personality Disorders Unit (*n* = 9), the Emergency Outpatient Unit (*n* = 4), the Internet Psychiatry Unit (*n* = 4) and the Anxiety and Affective Disorders Unit (*n* = 2). Feasibility outcomes are summarized in Table 3 (standard program completion and module engagement; CSQ-8; NEQ-B; recruitment source counts) (Table 4).

**Table 4:** Adverse events (AE) reported via NEQ open-ended responses (*n* = 16).

Type of AE	n (%) of respondents	Perceived as caused by treatment
Increased stress/pressure	6 (38)	Yes/partly
Emotional strain	4 (25)	Yes
Negative self-view/alliance issues	1 (6)	Yes
Dependence/difficult after end	1 (6)	Yes
Worry/sleep disturbance	1 (6)	Partly (other factors also)
Dropout due to negative experience	1 (6)	Yes
Technical or form issues	1 (6)	Yes

*Note*. Percentages are based on participants who provided an open-ended response to NEQ (*n* = 16). AE = adverse event; NEQ = Negative Effects Questionnaire. Categories were divided inductively from responses. The column *Perceived as caused by treatment* reflects whether participants explicitly linked the AE to the intervention.

### 3.4 Exploratory symptoms outcomes

#### 3.4.1 Pre–post analyses

Pre–post outcome measures are summarized descriptively per instrument. Analyses were pairwise, meaning only participants with both pre- and posttreatment data on each measure were included. Descriptive results are reported in Table 5, Table 6. Across the full group (Table 5), effect size estimates indicated small to moderate reductions in depressive symptoms (*d* = −0.33), borderline symptom severity (*d* = −0.19), and emotion regulation difficulties (*d* = −0.32). Changes in anxiety, disability, and quality of life were minimal (*d* < 0.2).

**Table 5:** Pre- to posttreatment outcomes across the full sample (*N* varies by measure).

Measure	n	Pre M (SD)	Post M (SD)	Cohen's *d*
Emotion regulation				
DERS (Emotion Regulation)	46	54.22 (14.83)	50.72 (17.52)	−0.24
BSL-23 (Borderline Symptoms)	46	1.46 (0.84)	1.37 (0.89)	−0.10
ERQ total	26	3.78 (0.93)	3.77 (0.90)	−0.02

General symptoms				
PHQ-9 (Depression)	46	13.00 (5.80)	12.57 (6.26)	−0.07
GAD-7 (Anxiety)	42	10.19 (5.25)	10.00 (4.80)	−0.04

Function and health				
WHODAS (Disability)	45	27.11 (15.93)	26.20 (18.35)	−0.06
EQ-5D Index (Health Index)	45	0.75 (0.16)	0.74 (0.23)	−0.03
EQ-5D Self-Rated Health	44	53.43 (18.26)	51.86 (19.98)	−0.09

*Note*. Values are means (M) and standard deviations (SD). Cohen's *d* values are calculated using pre-treatment SD. Negative *d* indicates improvements for DERS, BSL-23, PHQ-9, GAD-7, ERQ Total and WHODAS, while positive *d* indicates improvements for EQ-5D. Analyses were conducted pairwise, such that only participants with both pre- and posttreatment data were included; N therefore varies across instruments.

**Table 6:** Pre- to posttreatment outcomes by diagnostic group (PD, PSP, MDG).

Measure	PD group			PSP group			MDG group		
	Pre M (SD)	Post M (SD)	Cohen's *d*	Pre M (SD)	Post M (SD)	Cohen's *d*	Pre M (SD)	Post M (SD)	Cohen's *d*
Emotion regulation									
DERS-16	53.67 (10.40) *n* = 9	47.67 (13.32) *n* = 9	−0.58	56.53 (17.24) *n* = 15	52.33 (19.68) *n* = 15	−0.24	52.86 (15.04) *n* = 22	50.86 (17.85) *n* = 22	−0.13
BSL-23	1.43 (0.70) *n* = 9	1.27 (0.67) *n* = 9	−0.24	1.46 (0.86) *n* = 15	1.25 (0.80) *n* = 15	−0.25	1.46 (0.92) *n* = 22	1.50 (1.04) *n* = 22	+0.05
ERQ Total	3.92 (1.26) *n* = 5	3.88 (1.40) *n* = 5	−0.03	3.20 (0.66) *n* = 5	3.56 (1.01) *n* = 5	+0.54	3.92 (0.87) *n* = 16	3.79 (0.73) *n* = 16	+0.05

General symptoms									
PHQ-9	12.44 (6.73) *n* = 9	12.89 (5.88) *n* = 9	+0.07	13.21 (5.77) *n* = 14	12.50 (6.19) *n* = 14	−0.12	13.09 (5.71) *n* = 23	12.48 (6.76) *n* = 23	−0.11
GAD-7	8.00 (4.85) *n* = 9	8.67 (4.18) *n* = 9	+0.14	11.77 (5.42) *n* = 13	11.62 (5.04) *n* = 13	−0.03	10.15 (5.19) *n* = 20	9.55 (4.85) *n* = 20	−0.12

Function and health									
WHODAS	28.44 (14.73) *n* = 9	24.78 (13.75) *n* = 9	−0.25	26.21 (13.12) *n* = 14	23.50 (16.51) *n* = 14	−0.21	27.14 (18.47) *n* = 22	28.50 (21.29) *n* = 22	+0.07
EQ-5D Index	0.73 (0.18) *n* = 8	0.80 (0.14) *n* = 8	+0.40	0.74 (0.19) *n* = 14	0.71 (0.30) *n* = 14	−0.19	0.75 (0.15) *n* = 23	0.74 (0.22) *n* = 23	−0.14
EQ-5D Self-Rated	43.75 (14.82) *n* = 9	47.50 (14.65) *n* = 9	+0.25	56.00 (14.87) *n* = 14	53.64 (22.15) *n* = 14	−0.16	55.32 (20.70) *n* = 22	52.32 (20.81) *n* = 22	−0.14

*Note*. Values are means (M) and standard deviations (SD). Cohen's d values are calculated using pre-treatment SD. Negative *d* indicates improvements for DERS, BSL-23, PHQ-9, GAD-7, ERQ Total, and WHODAS, while positive *d* indicates improvements for EQ-5D. Analyses were conducted pairwise, such that only participants with both pre- and posttreatment data were included; N therefore varies across instruments.

By diagnostic group (Table 6), reductions in emotion regulation difficulties were observed in the PD (−5.05), PSP (−5.63), and MDG (−2.84) groups. Depression symptoms decreased in the MDG group (−1.87), the PSP group (−1.60), and the PD group (−0.59). Self-rated health (EQ–5D VAS) increased in the PD group (+4.0), decreased in the PSP group (−5.96), and was stable in the MDG group (−0.03).

##### 3.4.1.1 Emotion Regulation Questionnaire (ERQ)

ERQ scores were summarized descriptively because only a small number of participants completed the measures at posttreatment.

#### 3.4.2 Longitudinal analysis of CORE-10

**Fig. 2:**
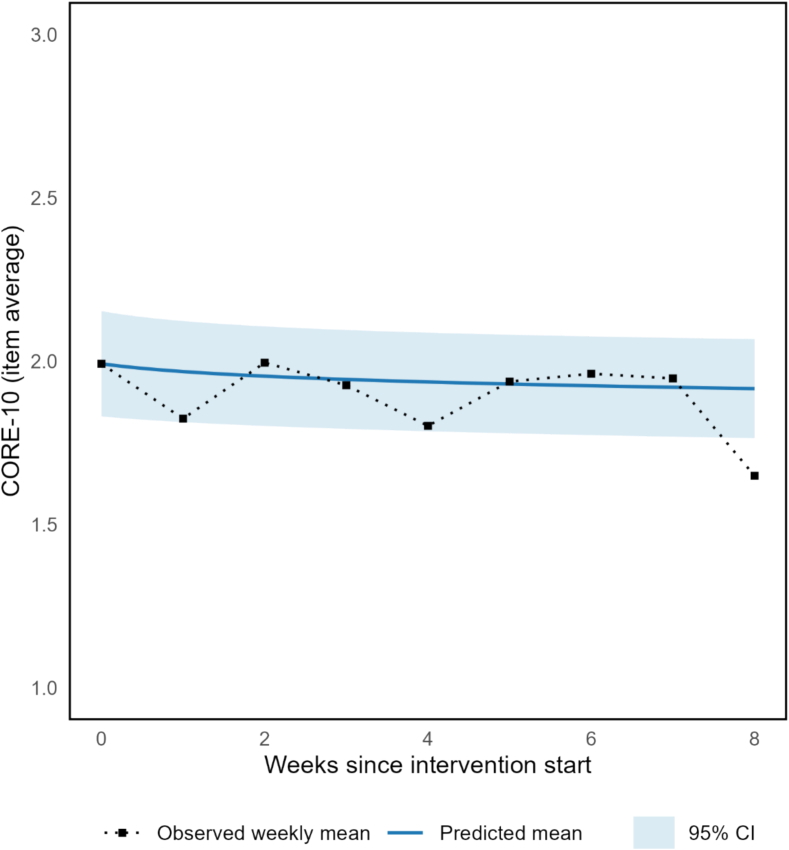
Observed and predicted CORE-10 scores over time. *Note.* Observed weekly CORE-10 scores (0–4 scale) and fixed-effect predictions from a linear mixed-effects model with log-transformed time; shaded areas represent 95% confidence intervals. The model showed a significant decrease over 8 weeks (*β* = −0.074, *p* = .031), based in 581 weekly observations.

Fig. 2 presents the observed and predicted CORE-10 trajectories over time. A linear mixed-effects model (LMM) with log-transformed calendar time as a fixed effect was estimated based on participants who engaged with the program, contributing to 581 observations. Results indicated a significant reduction in distress over time (*β* = −0.074, *SE* = 0.033, *t*(63.27) = −2.21, *p* = .031). Model comparison showed a marginally better fit for log-transformed time (AIC = 692.4) compared to session number (AIC = 693.5). Over a typical 8-week period, this corresponds to an estimated reduction of approximately 0.15 points on the CORE-10 mean scale (approximately 1.5 points on the 0–40 total score), suggesting a small average change at the group level. Inspection of the random effects indicated variability in both baseline and individual trajectories, with considerable heterogeneity across participants.

## 4 Discussion

This feasibility study evaluated the digital adaptation of Good Psychiatric Management (GPM-I) in a naturalistic setting. Accordingly, findings are interpreted to describe feasibility and acceptability in routine care, while symptom outcomes are reported as secondary and exploratory signals rather than evidence of effectiveness. Overall, the findings suggest that implementation is workable and may serve as a foundation for further development and evaluation.

### 4.1 Feasibility and acceptability

Of the 131 participants who were given access to the program, 78 initiated the intervention by completing the first module. Among initiators, 40/78 (51.3%) completed the standard program (Modules 1–2), and 13/78 (16.7%) completed all available modules (Modules 1–6), i.e., also completing the optional advanced modules (Modules 3–6). These completion rates should be interpreted in light of how completion is defined (module completion versus posttreatment assessment completion), and direct benchmarking against meta-analytic adherence estimates for guided digital interventions is limited by differences in populations, intervention formats, and completion definitions (van Ballegooijen et al., 2014). Accordingly, we refrain from making strong comparability claims. The relatively low rate of completion of all available modules (Modules 1–6) suggests that a six-module format may be demanding in routine care. In contrast, the higher completion rate for the standard program (Modules 1–2) supports treating Modules 3–6 as optional advanced modules rather than as an expected component of standard delivery. Importantly, engagement metrics should be interpreted at the level of routine-care feasibility rather than as establishing feasibility for personality pathology as the primary clinical concern.

Satisfaction ratings (CSQ–8) were moderate among those who responded: most reported satisfaction, willingness to return, and perceived benefit. However, only about one third of participants completed these assessments, which limits conclusions about overall acceptability. Therefore, satisfaction ratings likely reflect a selected subgroup and may overrepresent participants with more favorable experiences. Moderate ratings may partly relate to the program's structure, where the standard program (Modules 1–2) is followed by optional advanced modules (Modules 3–6), which may have increased perceived burden and reduce clarity around what constitutes completion. This selection may be partly driven by procedural factors, including optional posttreatment assessments and the separation between treatment delivery and outcome measurement platforms. Negative effects were reported by a similar proportion, typically mild to moderate in severity, most often increased stress or emotional strain.

Several contextual factors shaped feasibility. The sample was heterogeneous, including both personality disorders and personality difficulties with and without additional comorbidity. Notably, the largest group was the mixed diagnostic group (MDG), many of whom had neurodevelopmental disorders, particularly ADHD. In routine care, personality-related difficulties may be documented as secondary clinical features rather than as the primary diagnosis, which likely contributed to the size of the MDG subgroup. This raises questions about whether the format of GPM-I is equally suitable across groups, as the program requires sustained attention and executive functioning to process relatively text-heavy material, particularly given the large proportion of participants with neurodevelopmental disorders in the MDG group. In a text-based, self-directed format, these cognitive demands may contribute to early disengagement and may partly account for the observed drop-off after Module 1. For individuals with ADHD, this may present a barrier to engagement and suggest that further adaptation could be necessary. Potential adaptations include shorter text blocks, more visual and audio content, and increased scaffolding (e.g., summaries and prompts) to reduce executive load. At the same time, the PD and PSP groups together were of similar size to the MDG group, indicating that GPM-I reached both individuals with clinically established personality disorders and those with personality difficulties. However, given that nearly half of the sample belonged to the MDG subgroup, feasibility specifically for patients primarily presenting with personality pathology cannot be established from the present data and should be interpreted separately from routine-care feasibility across diagnostic presentations. Feasibility may also vary with cognitive and functional profiles (e.g., attention and executive functioning demands).

The design of GPM-I emphasized two main processes: psychoeducation about personality and emotions, and support for basic change efforts. Psychoeducational content was central to the first module and focused on increasing knowledge of how many everyday problems can be understood as difficulties in emotion regulation, alongside knowledge about how personality and genetic factors set the framework for who we are. Change efforts were encouraged throughout the program, with participants asked to take small weekly steps, and some implemented the change-plan provided in the final module. Therapist feedback was often extensive and personalized, yet responses from participants were limited. Although not systematically analyzed in the present study, written interaction between therapist and participant may represent a key process variable in the guided digital format of GPM-I, both as a marker of engagement and as a potential mechanism supporting change efforts. Future studies could operationalize this interaction (e.g., frequency, timing, and content of feedback and responses) to examine its association with module completion and symptom trajectories. The limited responsiveness also raised questions about participants' motivation, which may have been influenced by the fact that all were referred rather than self-referred, an aspect worth considering in future applications. This referral-based enrollment may also help explain the variability in engagement observed in this feasibility context. For some participants referred from the ADHD unit, GPM-I functioned as an interim option while awaiting other interventions. This may have influenced treatment preference and engagement; future studies should assess expectations and treatment preference at baseline.

### 4.2 Secondary outcomes

Symptom outcomes were secondary and exploratory. Weekly CORE-10 scores showed a small but statistically significant reduction in general psychological distress (*β* = −0.074, *p* = .031), consistent with the pattern illustrated in Fig. 2. Pre–post measures showed small and inconsistent changes across domains (Table 5, Table 6). Group–level differences were numerical only and did not reach statistical significance. Group-level patterns showed small numerical differences, but these were inconsistent, not statistically significant, and should not be interpreted beyond feasibility-level signal value. None of these group differences were significant, and in some cases baseline severity was higher in PSP than PD, underscoring the heterogeneity of the sample.

The reduction in CORE-10 scores is noteworthy given the heterogeneity of the sample. Beyond this finding, results were mixed, and the study was not designed to evaluate effectiveness. Accordingly, these outcomes should be regarded as preliminary signal data, with feasibility remaining the primary conclusion.

### 4.3 Limitations

Several methodological aspects should be considered when interpreting the findings, many of which also provide valuable information for future implementation.

#### 4.3.1 Design limitations

A central limitation is the mixed diagnostic composition of the sample, which constrains conclusions about feasibility for personality pathology as the primary clinical concern. In addition, the lack of a control group precludes causal inference regarding symptom change, and observed pre–post differences may reflect natural recovery, regression to the mean, or concurrent care. Small subgroups from several recruitment sources were aggregated due to low numbers (e.g., emergency services, the Affective and Anxiety Disorders Program, and Internet Psychiatry), limiting conclusions about feasibility across referral sources. Because these subgroups were too small to analyze meaningfully, aggregation may also conceal subgroup-specific implementation barriers.

#### 4.3.2 Implementation limitations

The most concrete limitation concerned how feasibility measures were administered. These questionnaires were optional and easy to skip, in contrast to symptom measures such as CORE-10, which were embedded within the treatment flow and required completion before proceeding. This design, together with pre–post assessments administered through a separate system outside the treatment platform, likely contributed to lower assessment completion rates. Low response rates on posttreatment acceptability measures further limit interpretability, as respondents may differ systematically from nonresponders in ways not captured by the available data. Accordingly, satisfaction outcomes should be interpreted cautiously and may not generalize to all participants.

Care pathways and concurrent treatments were heterogeneous (adjunct vs stand-alone while awaiting services), which complicates attribution of exploratory symptom change and may have influenced engagement and satisfaction. We had aimed to recruit larger numbers overall and within each care pathway/diagnostic subgroup to enable more informative subgroup analyses; however, subgroup sizes were too small for meaningful comparisons.

Therapist feedback and communication were not structured in a way that readily allows systematic analysis. Given that much of the therapeutic contact occurred through written exchanges, clearer routines for documentation would strengthen future evaluations. In addition, if eligibility criteria were applied with flexibility in individual cases as part of routine care decision-making, this may have affected internal validity and reduced comparability to a future controlled trial with stricter application of inclusion/exclusion criteria. Another limitation was that the study did not include specific measures targeting the primary aims of the intervention, namely increased understanding of personality functioning and personal change efforts such as setting and testing new behavioral goals. Developing and validating such measures could strengthen the evaluation of future versions of the program.

Taken together, these limitations reflect both constraints inherent to the feasibility design and several modifiable implementation targets, including refinement of the program's technical design, measurement strategy, and communications routines to better capture the processes it aims to promote.

### 4.4 Future directions

Future research should evaluate GPM-I in larger samples with more rigorous design. Incorporating measures of psychoeducational outcomes could test whether participants actually increased their knowledge in key areas. Systematic analyses of therapist–participant communication may also shed light on engagement processes. Selection procedures also deserve attention; voluntary self-enrollment may help identify participants with sufficient intrinsic motivation to benefit from the program. Future implementation efforts should examine how a digital format like GPM-I can function as a scalable option or complement to standard care, including optimization of module structure, platform usability, and assessment procedures.

### 4.5 Conclusion

This feasibility study provides useful feasibility signals that a digital adaptation of GPM can be delivered in routine psychiatric care, defined here as feasible recruitment/onboarding procedures, delivery within routine workflows, and no major safety concerns were indicated among respondents. Out of 131 participants given access, 78 initiated the program, with 40/78 (51.3%) completing the standard program (Modules 1–2), and 13/78 (16.7%) completing all available modules (Modules 1–6), suggesting moderate engagement. Satisfaction among respondents was generally moderate, but low response rates limit conclusions about overall acceptability. Overall, the findings mainly inform areas for refinement prior to a larger controlled trial, particularly regarding engagement supports and assessment adherence, while situating GPM-I within the broader ICD-11 framework as a low-threshold stepped-care option. Given the mixed diagnostic composition, findings should be interpreted as routine-care feasibility rather than establishing feasibility for personality pathology as the primary clinical concern.

## Declaration of competing interest

The authors declare that they have no known competing financial interests or personal relationships that could have appeared to influence the work reported in this paper.
